# Supplementary material for: Purchasing of tobacco-related and e-cigarette-related products within prisons before and after implementation of smoke-free prison policy: analysis of prisoner spend data across Scotland, UK
Source: BMJ Open. 2022 Feb 22;12(2):e058909. doi: 10.1136/bmjopen-2021-058909 (PMC8867351; doi:10.1136/bmjopen-2021-058909)
Supplement: Author's manuscript [file bmjopen-2021-058909.draft_revisions.pdf]

BMJ Open is committed to open peer review. As part of this commitment we make the peer review history of every article we publish publicly available.

When an article is published we post the peer reviewers' comments and the authors' responses online. We also post the versions of the paper that were used during peer review. These are the versions that the peer review comments apply to.

The versions of the paper that follow are the versions that were submitted during the peer review process. They are not the versions of record or the final published versions. They should not be cited or distributed as the published version of this manuscript.

BMJ Open is an open access journal and the full, final, typeset and author-corrected version of record of the manuscript is available on our site with no access controls, subscription charges or pay-per-view fees (http://bmjopen.bmj.com).

If you have any questions on BMJ Open's open peer review process please email
info.bmjopen@bmj.com

BMJ Open

Purchasing of tobacco-related and e-cigarette-related products within prisons before and after implementation of smoke-free prison policy: analysis of prisoner spend data across Scotland.

Journal:	*BMJ Open*
Manuscript ID	bmjopen-2021-058909
Article Type:	Original research
Date Submitted by the Author:	01-Nov-2021
Complete List of Authors:	Best, Catherine; University of Stirling, Faculty of Health Sciences and Sports<br>Brown, Ashley; University of Stirling, Institute for Social Marketing and Health<br>Hunt, Kate; University of Stirling, Institute for Social Marketing and Health
Keywords:	Health policy < HEALTH SERVICES ADMINISTRATION & MANAGEMENT, PUBLIC HEALTH, PREVENTIVE MEDICINE

SCHOLARONE™
Manuscripts

I, the Submitting Author has the right to grant and does grant on behalf of all authors of the Work (as defined in the below author licence), an exclusive licence and/or a non-exclusive licence for contributions from authors who are: i) UK Crown employees; ii) where BMJ has agreed a CC-BY licence shall apply, and/or iii) in accordance with the terms applicable for US Federal Government officers or employees acting as part of their official duties; on a worldwide, perpetual, irrevocable, royalty-free basis to BMJ Publishing Group Ltd ("BMJ") its licensees and where the relevant Journal is co-owned by BMJ to the co-owners of the Journal, to publish the Work in this journal and any other BMJ products and to exploit all rights, as set out in our licence.

The Submitting Author accepts and understands that any supply made under these terms is made by BMJ to the Submitting Author unless you are acting as an employee on behalf of your employer or a postgraduate student of an affiliated institution which is paying any applicable article publishing charge ("APC") for Open Access articles. Where the Submitting Author wishes to make the Work available on an Open Access basis (and intends to pay the relevant APC), the terms of reuse of such Open Access shall be governed by a Creative Commons licence – details of these licences and which Creative Commons licence will apply to this Work are set out in our licence referred to above.

Other than as permitted in any relevant BMJ Author's Self Archiving Policies, I confirm this Work has not been accepted for publication elsewhere, is not being considered for publication elsewhere and does not duplicate material already published. I confirm all authors consent to publication of this Work and authorise the granting of this licence.

For peer review only

Purchasing of tobacco-related and e-cigarette-related products within prisons before and after implementation of smoke-free prison policy: analysis of prisoner spend data across Scotland.

Corresponding author: Dr Catherine Best, G10 Pathfoot, Institute for Social Marketing and Health, Faculty of Health Sciences and Sport, University of Stirling, Stirling, UK. Email: catherine.best2@stir.ac.uk

Dr Ashley Brown, Institute for Social Marketing and Health, Faculty of Health Sciences and Sport, University of Stirling, Stirling, UK.

Professor Kate Hunt, Institute for Social Marketing and Health, Faculty of Health Sciences and Sport, University of Stirling, Stirling, UK.

Word count: 2917

For peer review only

For peer review only - http://bmjopen.bmj.com/site/about/guidelines.xhtml

Abstract

Introduction: Scotland implemented smoke-free policy throughout its prisons in November 2018. Provision of e-cigarettes through the prison 'canteen' (shop) was part of the implementation strategy. This study compares purchasing patterns (all products, nicotine-related products, and food and beverage products) in the prison canteen before and after implementation of smoke-free policy across a national prison system.

Methods: Canteen spend data were provided for the period July 2018-September 2019 by the Scottish Prison Service. In a series of generalised linear mixed effects models, the amount spent before and after implementation of smoke-free prison policy was compared for all purchases in the time period, and for people in custody identified as 'smokers' and 'non-smokers' from their pre-implementation tobacco purchasing patterns who were resident within prison before, during and after the implementation of smoke-free policy.

Results: The results of interrupted time-series analysis indicated that total canteen spend did not change over the study period. However, the amount spent on nicotine-related products significantly decreased from pre- to post-implementation. Post-implementation about 25% of previous 'smokers' total canteen spend was on nicotine-related products. The amount spent by previous 'smokers' on food and beverages increased post-implementation.

Conclusion: Although the amount of money previous 'smokers' in prison spent on nicotine-related products decreased after smoke-free policy, nicotine products still account for a large proportion of canteen spend among people in custody in smoke-free prisons in Scotland. Results indicate that many people in custody may continue to use nicotine in smoke-free prisons where e-cigarettes are permitted.

Strengths and Limitations

For peer review only - http://bmjopen.bmj.com/site/about/guidelines.xhtml

The strength of this study is that we use purchase data from all publically-managed closed prisons in Scotland to investigate the effects of smokefree prisons policy on purchases from the prison canteen (shop) by people in custody.

The main limitation of this study is that we were not able to link the purchase data to other information and therefore have to use purchase patterns (previous purchase of tobacco) as a proxy for smoking status.

For peer review only

For peer review only - http://bmjopen.bmj.com/site/about/guidelines.xhtml

Introduction

In the UK[1] and elsewhere[2], people who are or have been in prison experience worse health than the general population. The prison population is disproportionately drawn from marginalised sections of society; the imprisonment process can intensify or alleviate risk factors for poor health. For example, smoking rates are higher in socioeconomically deprived groups[3] and even higher amongst people in custody (PiC) in prisons where smoking is still permitted; in Scotland 68% of people in custody smoked in 2017[4], resulting in high levels of secondhand smoke (SHS)[5,6]. Due to concern about the effects of smoking on the health of PiC and occupational SHS exposure for staff, the sale and use of tobacco was prohibited in Scottish prisons from 30 November 2018.

While detained, basic food and toiletries are provided but PiC in Scotland can purchase additional food and beverage products, personal hygiene products and other items from lists for the 'canteen' (prison shop). In anticipation of the smoke-free policy, tobacco was removed from the canteen in mid-November 2018. As part of a comprehensive implementation plan, selected e-cigarette products were added to the canteen list to assist PiC in the transition to a tobacco-free environment; some PiC were eligible for free 'starter' packs provided in November 2018.[7]

To date, implementation of smoke-free prisons has not included the provision of e-cigarettes in jurisdictions outside of the UK, although e-cigarettes have subsequently gone on sale in some US prisons which are smoke-free. Scotland was the first country to undertake a multi-phase, multi-methods research programme to evaluate the process and outcomes of smoke-free prison policy[8], including immediate and longer term impacts on levels of PM2.5 as a measure of SHS exposure[6][9] and the response of staff and PiC to smoke-free policy before [5][10] and after implementation[11]. The prison canteen is important for PiC, since, for example, it is a means through which they can purchase toiletries, which can enhance their sense of dignity in a potentially challenging environment, or phone cards, which enable them to keep in better touch with family and friends. The proportion of their limited available resources which are spent on nicotine-related products

For peer review only - http://bmjopen.bmj.com/site/about/guidelines.xhtml

(tobacco-related and e-cigarette-related products, where allowed) in the canteen thus potentially has a large impact on the finances, resources and wellbeing in this vulnerable population. It is important to understand whether PiC spend less or more on e-cigarette products in the post-'ban' period than they did previously on tobacco, to inform understandings of the individual and wider public health consequences of smoke-free prison policies. Effects on broader patterns of prison canteen purchasing behaviour (e.g. changes in spend on confectionery or sugar-sweetened beverages (SSBs)) may signal other unintended (positive or negative) impacts of smoke-free prison policy, with potential implications for levels of obesity within prisons. In Canada , Johnson and colleagues found that smoke-free prison policy was associated with weight gain among PiC [12] and that weight gain while in prison was more closely correlated with the type of food purchased from the canteen than with food provided to PiC.[13] Any increases in purchases of obesogenic food products following the removal of tobacco from prisons may be an important consideration for other jurisdictions preparing to implement smoke-free prison policy.

Aim

This study examines purchasing data from the prison canteen in all 12 closed publicly-run prisons in Scotland before and after implementation of smoke-free prison policy in 2018.

Methods

The prison population in Scotland was around 8200 people in July 2019. 79% of the prison population had been sentenced and the remainder were untried or awaiting sentence.[14] Anonymised data on individuals' purchases were supplied by Scottish Prison Service (SPS). PiC within 12 of the 15 prisons in Scotland (i.e. excluding the one publicly-run 'open' and two privately-run prisons) in the form of de-identified, password protected excel worksheets. The data set received

For peer review only - http://bmjopen.bmj.com/site/about/guidelines.xhtml

from SPS comprised information for purchases made between 18th July 2018, one year after the SPS announced its intention to make all prisons smoke-free in November 2018, and 30th September 2019.

We coded every purchase into one of six product types: *tobacco-related* (including tobacco, cigarette papers, filters, lighters and cigarette rolling devices); *e-cigarette-related* (devices, e-liquids and chargers); *personal hygiene*; *food* (including sweets, snacks and beverages); *communications* (stamps, writing materials and phone cards); or *other*. We then calculated the total spend for each individual per week on all products combined and for each of these six product types. Finally, we created a combined measure of the total spend on any tobacco-related or e-cigarette-related products by each person, per week, referred to collectively hereafter as 'nicotine-related products'. These include manufactured cigarettes, rolling tobacco, papers, filters and other smoking paraphernalia and e-cigarette products such as devices, e-liquid refills, and chargers. Nicotine Replacement Therapy was not included as although it was available through the canteen purchase levels were so low as to be negligible.

Ethical Approval

This study was approved by the General University Research Ethics Panel of the University of Stirling ref GUEP497(A).

Patient and Public Involvement

It was not possible to involve study participants in the design, or conduct, or reporting, or dissemination plans of our research.

Analysis

We conducted a series of generalised linear mixed effects models to examine changes in the total spend in the canteen per week and on the amount spent on nicotine-related products over time. These models account for the dependencies among repeated measures for each individual over time

For peer review only - http://bmjopen.bmj.com/site/about/guidelines.xhtml

by including an individual level random intercept. The effect of the implementation of smoke-free prison policy on 'canteen' purchasing behaviour, expressed as incident rate ratios, was estimated using an interrupted time series approach. The time series was divided into three periods:

1. the period before the removal of tobacco products from the canteen list (i.e. Period 1 29/7/2018-30/9/2018)
2. the period including the introduction of rechargeable e-cigarette products and removal of tobacco-related products (i.e. Period 2 1/10/2018-31/1/2019).
3. the period after these changes were made (i.e. Period 3 1/2/2019-31/3/2019).

The comparison of interest was between Periods 1 and 3. The estimate of effects of Period 2 versus Period 1 was of less interest as there were several changes during this time related to the imminent implementation of smoke-free policy, including the addition of two brands of rechargeable e-cigarettes to the canteen list, the provision of free introductory e-cigarette packs to eligible, recorded smokers and a graded pricing structure for e-cigarette products during this period (see Brown et al [10] for more detail). Period 2 also includes the Christmas period, when canteen spending patterns are temporarily disrupted. Hence, the comparison of Period 1 versus Period 3 best captures the effect of the smoke-free policy and the introduction of rechargeable e-cigarette products in Scottish prisons.

The generalised mixed models included a factor variable for prison, a variable for week number (1 to 35), a factor variable for time period, and an interaction between the two. The distribution of the purchase data was highly skewed with lots of small purchases and relatively few high value purchases. The appropriate link function for the generalised linear model was determined by comparing the Akaike information criterion (AIC) for mixed effects Poisson, negative binomial and linear models. For all analyses the negative binomial model (log link) was associated with the smallest information loss. Marginal effects for the implementation of smoke-free prison policy were

For peer review only - http://bmjopen.bmj.com/site/about/guidelines.xhtml

computed for all models. Marginal effects are a way of demonstrating the impact of change in one variable (pre-implementation versus post-implementation) based on a model containing features such as interactions and random effects.

We also sought to take account of smoking status and the considerable turnover in the prisoner population over time. To account for effects of turnover in prison population we conducted an analysis including only people who had made purchases in periods 1-3. In addition, in order to distinguish the impact on likely smokers and likely non-smokers amongst PiC prior to implementation, we identified those who had made any purchase of tobacco in Period 1 as 'smokers'.

To understand overall canteen spend, and changes in purchasing over time, we therefore conducted analyses in three different samples:

1. Group 1 - the full dataset of all purchases;
2. Group 2 - people who had purchased tobacco before implementation ('smokers' in Period 1) and made purchases through Periods 2 and 3 (i.e. those who were still imprisoned);
3. Group 3 - people who had not purchased tobacco before implementation ('non-smokers in' Period 1) who made purchases through Period 2 and 3.

As there were a very large number of observations included in the analyses, the statistical significance threshold was set at 0.01. Data management and cleaning were conducted in Python and data analysis in Stata version 15; graphs were generated in R (v 4.0.3).

Results

Products

For peer review only - http://bmjopen.bmj.com/site/about/guidelines.xhtml

For peer review only

Tobacco-related products pre-implementation

The total range of tobacco-related products sold in the pre-implementation period is shown in Table 1. For each product, the unit price, the total spent and the total number of units purchased within Period 1 are also presented. No information is presented for the post-implementation period, as no tobacco-related products were sold via the canteen during this period.

Of the 21 unique tobacco-related products available for purchase in the pre-implementation period, spending was heavily concentrated on a small number of products and one brand ('Brand 2') of rolling tobacco accounted for 48% (185175/387608.61) of all spending on tobacco-related products in Period 1.

E-cigarette-related products

Table 2 shows the price per item, total amount spent and the quantity of items purchased for all e-cigarette-related products for sale via the prison canteen in the time periods of interest (Period 1 and Period 3). Sixteen e-cigarette-related products were available in the post-implementation period (those not yet available in Period 1 are shown as £0 spend in Table 2). Again, most purchasing was concentrated on a small number of products. The most popular product was a tobacco-flavoured e-liquids at 18mg strength ('Brand A'), which alone accounted for 53% (149795.20/280257.92) of all spending on e-cigarette-related products.

Median spend per person per week by product type

Figure 1 shows the median spend per person per week for the six purchasing categories (tobacco-related, e-cigarette-related, personal hygiene, food, communications and other). The first graph

For peer review only - http://bmjopen.bmj.com/site/about/guidelines.xhtml

shows the composition of canteen spend for all people in the dataset (n=11944), the second graph is for 'smokers' who were present in the dataset in all three periods (n=2541) and the third is for 'non-smokers' present across all the three time periods (n=342).

The dotted lines in Figure 1 indicate the date of the introduction of smoke-free policy in Scottish prisons. The yellow shading indicates Period 2 which included the one-off provision of free e-cigarette starter packs for eligible smokers; changes in purchasing patterns during the Christmas period are clearly seen in the spike in purchases in late December.

Analysis of changes in canteen purchasing behaviour

We examined the relationship between the implementation of smoke-free prison policy and weekly canteen spend by individuals for three dependent variables, i.e. individual canteen spend per week on: all products; nicotine-related products; and food and beverage products.

We examined these outcomes for: i) the full sample, i.e. all people who purchased anything from the canteen during the study period (n=11994), ii) a) 'smokers' and b) 'non-smokers' making purchases throughout the implementation period (Periods 1, 2 and 3) defined as purchasing in at least 31 of 35 weeks (n=2541). Table 3 shows results for three groups.

The results in Table 3 indicate that there was no notable change in the total spend per person per week on canteen products (IRR 0.92 99% CI 0.84 to 1.00) following implementation of smoke-free prison policy: the marginal predicted total canteen spend per person per week in the full sample in the pre-implementation period was £20.46 (99% CI 19.98 to 20.94) and the marginal predicted spend in the post-implementation period was £19.43 (99% CI 18.88 to 19.98).

For peer review only - http://bmjopen.bmj.com/site/about/guidelines.xhtml

The implementation of smoke-free policy was associated with a decrease in the amount spent per week on nicotine-related products or NVPs ($p<0.001$). This was evident in both the full sample (Group 1) and those who were identified as ‘smokers’ in the pre-implementation period and were resident in prison for the full period under study (Periods 1-3) (Group 2). The marginal predicted spend on nicotine-related products in the pre-implementation period in these ‘smokers’ was £9.23 (99% CI 8.35 to 10.12) and in the post-implementation period was £5.21 (99% CI 4.66 to 5.77). In the post-implementation period (Period 3) the amount individuals spent on nicotine-related products showed a slight statistically significant week by week increase.

There was no change in the amount spent on nicotine-related products by people designated as ‘non-smokers’. That is, there is no indication people who had *not* purchased tobacco during Period 1 had started purchasing e-cigarette-related products in the post-implementation period.

In contrast, the amount spent on food and beverage products increased in the post-implementation period. This effect was only significant in Group 2 (‘smokers’ who were resident in the prison over for the full period under study (Periods 1-3)). The marginal predicted spend on food in the pre-implementation period by these ‘smokers’ increased from £8.67 (99% CI 8.23 to 9.13) to £10.24 in the post-implementation period (99% CI 9.58 to 10.90).

Discussion

This study has shown that on average, following the introduction of smoke-free prison policy, people in custody in Scotland spend less per week on canteen purchases for e-cigarette-related products than they previously did on tobacco-related products. The incident rate ratio for the change is 0.35 (99% CI 0.25 to 0 .50). This decline is also evident in total spend on tobacco-related and e-cigarette-related products at £393,843.45 and £280,257.92 in the pre- and post-implementation periods respectively. Nevertheless, the amount spent on nicotine-related products still represents a large

For peer review only - http://bmjopen.bmj.com/site/about/guidelines.xhtml

proportion of the total amount spent in the canteen by PiC, with most previous 'smokers' spending around 25% of their total spend per week in the canteen on e-cigarette-related products following implementation of smoke-free policy. This concurs with findings from qualitative interviews with PiC in Scotland. On the positive side, interviewees who were spending less on e-cigarettes post-implementation of smoke-free policy than they had previously spent on tobacco described benefits such as being able to buy healthier food from the canteen or other valued items, or being able to save money. However, there were beliefs that e-cigarettes were not affordable relative to the income for PiC with the fewest resources. Additionally, some complaints were made about the high upfront costs of vaping in prison and that the e-cigarette-related products available on the canteen list at that time represented poor value for money e.g. because they were perceived as less satisfying than conventional cigarettes or as compared to costs of e-cigarette products in the community.[7]

The amount spent on food and beverages by previous 'smokers' increased in the post-implementation period relative to pre-implementation The incident rate ratio for the change was 1.24 (99% CI 1.08 to 1.43). This possibly indicates snacking was used as a displacement activity following the removal of tobacco[15,16] and/or that people were able to diversify their discretionary food purchases due to saving money on nicotine-related products. Further research is required to examine differences in the breakdown of the food purchasing activity within food groups pre- and post-implementation, and whether this impacts on body mass index and other health-related indicators.

We found that although a range of tobacco products were available pre-implementation, purchasing was concentrated in a very small number of products, dominated by rolling tobacco. Post-implementation, nicotine-related purchasing was also concentrated on a narrow range of products, e.g., the most popular being the cheapest and highest strength e-liquid (18mg/ml). This possibly indicates that previous smokers were trying to maximise nicotine intake while minimising spending.

For peer review only - http://bmjopen.bmj.com/site/about/guidelines.xhtml

Limitations

It was not possible to link the canteen data to other sources of information about individuals (e.g. age, gender, custody status, smoking status) and we were therefore restricted to making assumption about whether individuals were smokers or not based on their purchasing patterns pre-implementation. We were also not able to link to information on smoking cessation support or the provision of prescribed NRT within the prisons' smoking cessation services.

There are limitations on the total amount of money people were permitted to spend on e-cigarettes post-implementation, set at the discretion of prison Governors. Limited information is available on these limits at individual prisons, but we understand that the guidance to Governors on limiting e-cigarette purchasing was far in excess of the median spend on e-cigarette products observed here.

In addition, our conclusions are limited to the amount of money that PiC spent on tobacco and e-cigarettes pre and post implementation. To date, we have not attempted to estimate how much nicotine was consumed and whether this differed from pre to post implementation. This is because the amount of nicotine that becomes bio-available varies enormously across products and methods of use.

The strengths of this study are that we have complete data from the canteen spend in closed publicly operated prisons in Scotland for a time period spanning the introduction of smoke-free prison policy. This is unique data and we are not aware of any other studies within the UK or internationally that have been able to study the spending choices of people in custody or to evaluate the effects of smoke-free prison policy on canteen spending.

Conclusion

For peer review only - http://bmjopen.bmj.com/site/about/guidelines.xhtml

Although smoke-free policy has decreased the amount of money previous 'smokers' in prison spend on nicotine-related products after implementation of smoke-free policy, nicotine-related products still account for a large proportion of discretionary purchases by people in custody. Ongoing monitoring is required to understand the longer-term benefits and risks of selling e-cigarettes in prisons, including how vaping affects the personal finances and health of people in custody and influences smoking behaviour post release.

References

1 Graham L, Fischbacher CM, Stockton D, *et al.* Understanding extreme mortality among prisoners: a national cohort study in Scotland using data linkage. *Eur J Public Health* 2015;**25**:879–85. doi:10.1093/eurpub/cku252

2 Dumont DM, Brockmann B, Dickman S, *et al.* Public health and the epidemic of incarceration. *Annu Rev Public Health* 2012;**33**:325–39. doi:10.1146/annurev-publhealth-031811-124614

3 Hiscock R, Bauld L, Amos A, *et al.* Socioeconomic status and smoking: a review. *Ann N Y Acad Sci* 2012;**1248**:107–23. doi:10.1111/j.1749-6632.2011.06202.x

4 Carnie J, Cameron J, Downie D, *et al.* Prisoner survey 2017 16. Edinburgh: 2017.

5 Sweeting H, Semple S, Demou E, *et al.* Predictors of opinions on prison smoking bans: Analyses of survey data from Scottish staff and prisoners. *Tob Induc Dis* 2019;**17**:47. doi:10.18332/tid/109559

6 Semple S, Dobson R, Sweeting H, *et al.* The impact of implementation of a national smoke-

For peer review only - http://bmjopen.bmj.com/site/about/guidelines.xhtml

free prisons policy on indoor air quality: results from the Tobacco in Prisons study. *Tob Control* 2020;**29**:234–6. doi:10.1136/tobaccocontrol-2018-054895

7 Brown A, O'Donnell R, Eadie D, *et al.* E-cigarette use in prisons with recently established smokefree policies: a qualitative interview study with people in custody in Scotland. *Nicotine Tob Res Off J Soc Res Nicotine Tob* Published Online First: December 2020. doi:10.1093/ntr/ntaa271

8 Hunt K, Semple S, Demou E, *et al.* Evaluating graduated progress towards and impacts of the implementation of indoor smoke free prison facilities in Scotland. *Public Heal Res* 2021;**in press**.https://www.journalslibrary.nihr.ac.uk/programmes/phr/155544/#/

9 Demou E, Dobson R, Sweeting H, *et al.* From Smoking-Permitted to Smokefree Prisons: A 3-Year Evaluation of the Changes in Occupational Exposure to Second-Hand Smoke Across a National Prison System. *Ann Work Expo Heal* 2020;**64**:959–69. doi:10.1093/annweh/wxaa073

10 Brown A, Sweeting H, Semple S, *et al.* Views of prison staff in Scotland on the potential benefits and risks of e-cigarettes in smoke-free prisons: a qualitative focus group study. *BMJ Open* 2019;**9**:e027799. doi:10.1136/bmjopen-2018-027799

11 Sweeting H, Demou E, Brown A, *et al.* Prisoners and prison staff express increased support for prison smoking bans following implementation across Scotland: results from the Tobacco In Prisons study. Tob. Control. 2020. doi:10.1136/tobaccocontrol-2020-055683

12 Johnson C, Chaput J-P, Diasparra M, *et al.* How did the tobacco ban increase inmates' body weight during incarceration in Canadian federal penitentiaries? A cohort study. *BMJ Open* 2019;**9**:e024552. doi:10.1136/bmjopen-2018-024552

13 Johnson C, Chaput J-P, Rioux F, *et al.* An exploration of reported food intake among inmates who gained body weight during incarceration in Canadian federal penitentiaries. *PLoS One* 2018;**13**:e0208768. doi:10.1371/journal.pone.0208768

For peer review only - http://bmjopen.bmj.com/site/about/guidelines.xhtml

14 Scottish Government. Scottish prison population: statistics 2019 to 2020. Edinburgh: 2020. https://www.gov.scot/publications/scottish-prison-population-statistics-2019-20/pages/1/

15 Spring B, Pagoto S, McChargue D, *et al.* Altered reward value of carbohydrate snacks for female smokers withdrawn from nicotine. *Pharmacol Biochem Behav* 2003;**76**:351–60. doi:10.1016/j.pbb.2003.08.008

16 Donny EC, Caggiula AR, Weaver MT, *et al.* The reinforcement-enhancing effects of nicotine: implications for the relationship between smoking, eating and weight. *Physiol Behav* 2011;**104**:143–8. doi:10.1016/j.physbeh.2011.04.043

For peer review only

Acknowledgements

We are grateful to staff at the Scottish prison service who assisted with the study and facilitated access to data. We are also grateful to the wider Tobacco In Prisons Study research team.

Funding

The study was supported by Cancer Research UK (C45874/A27016).

Competing interests

The authors have no competing interests to declare.

For peer review only - http://bmjopen.bmj.com/site/about/guidelines.xhtml

Table 1 Tobacco products available for purchase from the canteen list prior to implementation of smoke-free policy

		Pre-implementation period (Period 1)	
**Product**	Price (£)	Total spend 29/7/2018-30/9/2018	Total quantity 29/7/2018-30/9/2018
Pipe tobaccos			
**Brand 1 pipe tobacco 12.5g**	2.59	83400.59	32201
**Brand 9 pipe tobacco 50g**	10.20	408.00	40
**Brand 7 pipe tobacco 25g**	6.60	3042.60	461
Rolling tobaccos			
**Brand 2 rolling tobacco 30g**	12.50	185175.00	14814
**Brand 5 rolling tobacco 30g**	12.90	838.50	65
**Brand 6 rolling tobacco 30g**	13.35	1174.80	88

For peer review only - http://bmjopen.bmj.com/site/about/guidelines.xhtml

For peer review only

**Brand 10 rolling tobacco 30g**	10.00	32450.00	3245
**Brand 8 rolling tobacco 30g**	11.35	66318.05	5843
Factory made cigarettes/cigars			
**Brand 3 king size 20 cigarettes**	9.60	2611.2	272
**Brand 8 20 cigarettes**	9.65	434.25	45
**Brand 4 cigar drum**	1.01	131.30	130
Paraphernalia			
**Disposable lighter**	0.10	2554.10	25543
**RYO papers**	0.10-0.26	6725.94	54937
**Filters**	0.40-0.58	2329.70	4069
**Rolling machine**	1.62	14.58	9
**Total**		387608.61	

For peer review only - http://bmjopen.bmj.com/site/about/guidelines.xhtml

Table 2 Spend on e-cigarette-related Products pre- and post-implementation of smoke-free policy

	Pre-implementation (Period 1)			Post-implementation (Period 3)		
**Product**	Price	Total spend 29/7/2018 - 30/9/2018	Total quantity 29/7/2018 - 30/9/2018	Price	Total spend 1/2/2019-31/3/2019	Total quantity 1/2/2019-31/3/2019
Devices						
**Brand A rechargeable device**	7.00	574.00	82	7.00	5565.00	795
**Brand B rechargeable device**	4.33	329.08	76	6.49	1090.32	168
**USB mains plug for rechargeable device**	4.00	216.00	54	4.00	1048	262
**Introductory e-cigarette pack**	0	0	0	7.10	1874.40	264
**Disposable e-cigarettes**	1.20	3554.20	1594	1.20	4465.80	3673
Capsules						

For peer review only - http://bmjopen.bmj.com/site/about/guidelines.xhtml

**Brand A capsule – tobacco flavour 18mg**	3.20	246.40	77	3.20	149795.20	46811
**Brand A capsule berrymint - 12mg**	3.20	320.00	100	3.20	44422.40	13882
**Brand A capsule menthol - 12mg**	3.20	99.20	31	3.20	24902.40	7782
**Brand A capsule strawberry- 12mg**	3.20	358.40	112	3.20	13129.60	4103
**Brand A capsule red cherry - 12mg**	0	0	0	3.20	10665.60	3333
**Brand A capsule berry mint - 6mg**	0	0	0	3.20	451.20	141
**Brand B capsule strawberry 18mg**	6.80	292.40	43	6.80	7718	1135
**Brand B capsule blackcurrant - 18MG**	6.80	244.80	36	6.80	15130	2225
**Total**		6234.84			280257.92	

For peer review only

Table 3. Results from mixed effects negative binomial regression examining changes in canteen spend pre- versus post-implementation of smoke-free prison policy

		**Total spend (£)**	**Nicotine-related products (£)**	**Food items (£)**
	Variable	IRR (99% CI)	IRR (99% CI)	IRR (99% CI)
**Full sample n=11944 (Group 1)**	Pre vs post change	0.92 (0.84 to 1.00)	0.40 (0.33 to 0.51)**	1.12 (0.99 to 1.27)
	Change in time trend	1.00 (1.00 to 1.00)	1.02 (1.01 to 1.03)**	1.00 (1.00 to 1.01)
	Pre time trend	1.00 (1.00 to 1.00)	0.99 (0.99 to 1.00)	1.00 (0.99 to 1.00)
**'Smokers' resident within a prison for more than 31 weeks (Periods 1-3) n=2541 (Group 2)**	Pre vs post change	0.91 (0.81 to 1.03)	0.35 (0.25 to 0 .50)**	1.24 (1.08 to 1.43)*
	Change in time trend	1.00 (1.00 to 1.01)	1.02 (1.01 to 1.04)**	1.00 (0.99 to 1.00)
	Pre time trend	1.00 (1.00 to 1.00)	0.99 (0.99 to 1.00)	1.00 (1.00 to 1.00)
**'Non-smokers' resident within a prison for more**	Pre vs post change	0.87 (0.66 to 1.16)	0.08 (0.00 to 1.62)	0.80 (0.540 to 1.19)

For peer review only

For peer review only - http://bmjopen.bmj.com/site/about/guidelines.xhtml

**than 31 weeks n=342 (Group 3)**	Change in time trend	1.01 (0.99 to 1.02)	1.18 (1.03 to 1.36)	1.01 (0.99 to 1.02)
	Pre time trend	1.00 (0.99 to 1.01)	1.03 (0.93 to 1.14)	1.00 (0.99 to 1.01)

IRR= incident rate ratio * p<0.01 ** p<0.00

Figure 1 Median weekly spend per person by product category.

For peer review only

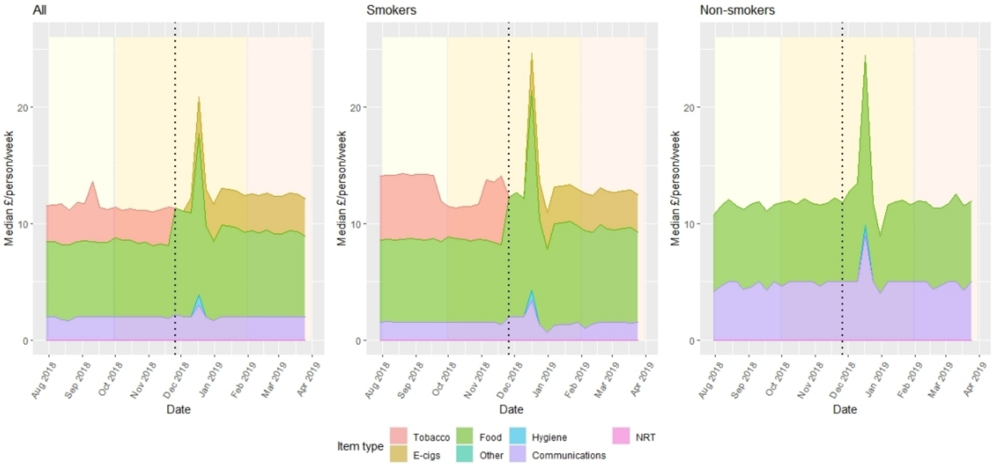

451x212mm (72 x 72 DPI)

STROBE Statement—Checklist of items that should be included in reports of ***cohort studies***

	Item No	Recommendation	Page No
**Title and abstract**	1	(*a*) Indicate the study's design with a commonly used term in the title or the abstract	1
		(*b*) Provide in the abstract an informative and balanced summary of what was done and what was found	1
**Introduction**			
Background/rationale	2	Explain the scientific background and rationale for the investigation being reported	2
Objectives	3	State specific objectives, including any prespecified hypotheses	2
**Methods**			
Study design	4	Present key elements of study design early in the paper	3
Setting	5	Describe the setting, locations, and relevant dates, including periods of recruitment, exposure, follow-up, and data collection	3
Participants	6	(*a*) Give the eligibility criteria, and the sources and methods of selection of participants. Describe methods of follow-up	3
		(*b*) For matched studies, give matching criteria and number of exposed and unexposed	
Variables	7	Clearly define all outcomes, exposures, predictors, potential confounders, and effect modifiers. Give diagnostic criteria, if applicable	3
Data sources/ measurement	8*	For each variable of interest, give sources of data and details of methods of assessment (measurement). Describe comparability of assessment methods if there is more than one group	4
Bias	9	Describe any efforts to address potential sources of bias	3
Study size	10	Explain how the study size was arrived at	4
Quantitative variables	11	Explain how quantitative variables were handled in the analyses. If applicable, describe which groupings were chosen and why	4
Statistical methods	12	(*a*) Describe all statistical methods, including those used to control for confounding	4
		(*b*) Describe any methods used to examine subgroups and interactions	
		(*c*) Explain how missing data were addressed	
		(*d*) If applicable, explain how loss to follow-up was addressed	
		(*e*) Describe any sensitivity analyses	
**Results**			
Participants	13*	(a) Report numbers of individuals at each stage of study—eg numbers potentially eligible, examined for eligibility, confirmed eligible, included in the study, completing follow-up, and analysed	4
		(b) Give reasons for non-participation at each stage	
		(c) Consider use of a flow diagram	
Descriptive data	14*	(a) Give characteristics of study participants (eg demographic, clinical, social) and information on exposures and potential confounders	4
		(b) Indicate number of participants with missing data for each variable of interest	
		(c) Summarise follow-up time (eg, average and total amount)	
Outcome data	15*	Report numbers of outcome events or summary measures over time	4, 5, 6

For peer review only

Main results	16	(*a*) Give unadjusted estimates and, if applicable, confounder-adjusted estimates and their precision (eg, 95% confidence interval). Make clear which confounders were adjusted for and why they were included<br>(*b*) Report category boundaries when continuous variables were categorized<br>(*c*) If relevant, consider translating estimates of relative risk into absolute risk for a meaningful time period	4 & Table 2
Other analyses	17	Report other analyses done—eg analyses of subgroups and interactions, and sensitivity analyses	6 &7
**Discussion**			
Key results	18	Summarise key results with reference to study objectives	7
Limitations	19	Discuss limitations of the study, taking into account sources of potential bias or imprecision. Discuss both direction and magnitude of any potential bias	7
Interpretation	20	Give a cautious overall interpretation of results considering objectives, limitations, multiplicity of analyses, results from similar studies, and other relevant evidence	7
Generalisability	21	Discuss the generalisability (external validity) of the study results	7
**Other information**			
Funding	22	Give the source of funding and the role of the funders for the present study and, if applicable, for the original study on which the present article is based	In electronic submission

*Give information separately for exposed and unexposed groups.

Note: An Explanation and Elaboration article discusses each checklist item and gives methodological background and published examples of transparent reporting. The STROBE checklist is best used in conjunction with this article (freely available on the Web sites of PLoS Medicine at http://www.plosmedicine.org/, Annals of Internal Medicine at http://www.annals.org/, and Epidemiology at http://www.epidem.com/). Information on the STROBE Initiative is available at http://www.strobe-statement.org.

For peer review only

For peer review only - http://bmjopen.bmj.com/site/about/guidelines.xhtml

BMJ Open

Purchasing of tobacco-related and e-cigarette-related products within prisons before and after implementation of smoke-free prison policy: analysis of prisoner spend data across Scotland, UK

Journal:	*BMJ Open*
Manuscript ID	bmjopen-2021-058909.R1
Article Type:	Original research
Date Submitted by the Author:	18-Jan-2022

Complete List of Authors:	Best, Catherine; University of Stirling, Faculty of Health Sciences and Sports<br>Brown, Ashley; University of Stirling, Institute for Social Marketing and Health<br>Hunt, Kate; University of Stirling, Institute for Social Marketing and Health

**Primary Subject Heading**:	Public health
Secondary Subject Heading:	Smoking and tobacco, Health policy
Keywords:	Health policy < HEALTH SERVICES ADMINISTRATION & MANAGEMENT, PUBLIC HEALTH, PREVENTIVE MEDICINE

SCHOLARONE™ Manuscripts

I, the Submitting Author has the right to grant and does grant on behalf of all authors of the Work (as defined in the below author licence), an exclusive licence and/or a non-exclusive licence for contributions from authors who are: i) UK Crown employees; ii) where BMJ has agreed a CC-BY licence shall apply, and/or iii) in accordance with the terms applicable for US Federal Government officers or employees acting as part of their official duties; on a worldwide, perpetual, irrevocable, royalty-free basis to BMJ Publishing Group Ltd ("BMJ") its licensees and where the relevant Journal is co-owned by BMJ to the co-owners of the Journal, to publish the Work in this journal and any other BMJ products and to exploit all rights, as set out in our licence.

The Submitting Author accepts and understands that any supply made under these terms is made by BMJ to the Submitting Author unless you are acting as an employee on behalf of your employer or a postgraduate student of an affiliated institution which is paying any applicable article publishing charge ("APC") for Open Access articles. Where the Submitting Author wishes to make the Work available on an Open Access basis (and intends to pay the relevant APC), the terms of reuse of such Open Access shall be governed by a Creative Commons licence – details of these licences and which Creative Commons licence will apply to this Work are set out in our licence referred to above.

Other than as permitted in any relevant BMJ Author's Self Archiving Policies, I confirm this Work has not been accepted for publication elsewhere, is not being considered for publication elsewhere and does not duplicate material already published. I confirm all authors consent to publication of this Work and authorise the granting of this licence.

For peer review only

Purchasing of tobacco-related and e-cigarette-related products within prisons before and after implementation of smoke-free prison policy: analysis of prisoner spend data across Scotland, UK

Corresponding author: Dr Catherine Best, G10 Pathfoot, Institute for Social Marketing and Health, Faculty of Health Sciences and Sport, University of Stirling, Stirling, UK. Email: catherine.best2@stir.ac.uk

Dr Ashley Brown, Institute for Social Marketing and Health, Faculty of Health Sciences and Sport, University of Stirling, Stirling, UK.

Professor Kate Hunt, Institute for Social Marketing and Health, Faculty of Health Sciences and Sport, University of Stirling, Stirling, UK.

Word count: 3304

For peer review only

Abstract

Objectives: To examine the effect of smoke-free prison policy implementation in November 2018 on purchasing patterns in the prison canteen (shop).

Design: Interrupted time series.

Setting: All 12 closed, publicly run prisons in Scotland, UK.

Participants: People in custody between August 2018 and end of March 2019 (n=11944).

Interventions: Implementation of smoke-free prisons policy

Outcome measures: Total spent on all products, nicotine-related products, and food and beverage products per week

Methods: Canteen data were provided for the period July 2018-September 2019 by the Scottish Prison Service. In a series of generalised linear mixed effects models, the amount spent before and after implementation of smoke-free prison policy was compared for all purchases in the time period, and for people in custody identified as 'smokers' and 'non-smokers' from their pre-implementation tobacco purchasing patterns.

Results: The amount spent on nicotine-related products significantly decreased from pre- to post-implementation (incident rate ratio [IRR] 0.40 99% CI 0.33 to 0.51, p<0.001). However, total canteen spend did not change over the study period (IRR 0.92 99% CI 0.84 to 1.00). Post-implementation about 25% of previous 'smokers' total canteen spend was on nicotine-related products. The amount spent by previous 'smokers' on food and beverages increased from £8.67 (99% CI 8.23 to 9.13) pre-implementation to £10.24 in the post-implementation period (99% CI 9.58 to 10.90).

Conclusion: Although the amount of money previous 'smokers' in prison spent on nicotine-related products decreased after smoke-free policy, nicotine products still account for a large proportion of canteen spend among people in custody in smoke-free prisons in Scotland. Results indicate that

For peer review only

For peer review only - http://bmjopen.bmj.com/site/about/guidelines.xhtml

many people in custody may continue to use nicotine in smoke-free prisons where e-cigarettes are permitted.

Strengths and limitations of this study

*A strength of this study is that we had a complete record of purchase data from all publicly managed closed prisons in Scotland, UK.

*The main limitation of this study is that we were not able to link the purchase data to other information and therefore have to use purchase patterns (previous purchase of tobacco) as a proxy for smoking status.

*Another limitation is that there were local restrictions on how much individuals could spend on e-cigarettes.

For peer review only - http://bmjopen.bmj.com/site/about/guidelines.xhtml

Introduction

In the UK [1] and elsewhere [2], people who are or have been in prison experience worse health than the general population. The prison population is disproportionately drawn from marginalised sections of society; the imprisonment process can intensify or alleviate risk factors for poor health. For example, smoking rates are higher in socioeconomically deprived groups[3] and even higher amongst people in custody (PiC) in prisons where smoking is still permitted; in Scotland 68% of people in custody smoked in 2017[4], resulting in high levels of secondhand smoke (SHS)[5,6]. Due to concern about the effects of smoking on the health of PiC and occupational SHS exposure for staff, the sale and use of tobacco was prohibited in Scottish prisons from 30 November 2018.

While detained, basic food and toiletries are provided but PiC in Scotland can purchase additional food and beverage products, personal hygiene products and other items from lists for the 'canteen' (prison shop); items are then delivered to someone's room (cell). People who are sentenced have the opportunity to make purchases from the canteen once per week. Spending limits apply, which vary depending on whether someone is on remand or convicted and their privilege level in prison. PiC who participate in employment or training in prison are paid a weekly wage (ranging from £5 to £21) [7], which can be spent in the canteen. Some PiC receive additional funds from family and friends. In anticipation of the smoke-free policy, tobacco was removed from the canteen in mid-November 2018. As part of a comprehensive implementation plan, selected e-cigarette products were added to the canteen list to assist PiC in the transition to a tobacco-free environment; some PiC were eligible for free 'starter' packs provided in November 2018.[8] Access to free combined behavioural and pharmacological treatments (e.g. NRT) continued to be available in prisons in the lead up to and following implementation of the smokefree policy [9].

To date, implementation of smoke-free prisons has not included the provision of e-cigarettes in jurisdictions outside of the UK, although e-cigarettes have subsequently gone on sale in some US prisons which are smoke-free. Scotland was the first country to undertake a multi-phase, multi-

For peer review only - http://bmjopen.bmj.com/site/about/guidelines.xhtml

methods research programme to evaluate the process and outcomes of smoke-free prison policy[8], including immediate and longer term impacts on air quality (measured as levels of fine inhalable particles with diameters of 2.5 micrometres and smaller - PM2.5)as a measure of SHS exposure[6][10] and the response of staff and PiC to smoke-free policy before [5, 11] and after implementation[12]. The prison canteen is important for PiC, since, for example, it is a means through which they can purchase toiletries, which can enhance their sense of dignity in a potentially challenging environment, or phone cards, which enable them to keep in better touch with family and friends. The proportion of their limited available resources which are spent on nicotine-related products (tobacco-related and e-cigarette-related products, where allowed) in the canteen thus potentially has a large impact on the finances, resources and wellbeing in this vulnerable population. It is important to understand whether PiC spend less or more on e-cigarette products in the post-'ban' period than they did previously on tobacco, to inform understandings of the individual and wider public health consequences of smoke-free prison policies. Effects on broader patterns of prison canteen purchasing behaviour (e.g. changes in spend on confectionery or sugar-sweetened beverages (SSBs)) may signal other unintended (positive or negative) impacts of smoke-free prison policy, with potential implications for levels of obesity within prisons. In Canada , Johnson and colleagues found that smoke-free prison policy was associated with weight gain among PiC [13] and that weight gain while in prison was more closely correlated with the type of food purchased from the canteen than with food provided to PiC.[14] Any increases in purchases of obesogenic food products following the removal of tobacco from prisons may be an important consideration for other jurisdictions preparing to implement smoke-free prison policy.

This study examines purchasing data from the prison canteen in all 12 closed publicly-run prisons in Scotland before and after implementation of smoke-free prison policy in 2018. The objective was to determine whether amount spent on all products, nicotine-related products, and food and beverage products from the prison canteen changed as a result of the implementation of smoke-free prisons policy. We examine these effects for all purchases in the time period, and then separately for those

For peer review only - http://bmjopen.bmj.com/site/about/guidelines.xhtml

people in custody identified as 'smokers' and 'non-smokers' from their pre-implementation tobacco purchasing patterns.

Methods

The prison population in Scotland was around 8200 people in July 2019. 79% of the prison population had been sentenced and the remainder were untried or awaiting sentence[15].Anonymised data on individuals' purchases were supplied by Scottish Prison Service (SPS). PiC within 12 of the 15 prisons in Scotland (i.e. excluding the one publicly-run 'open' and two privately-run prisons) in the form of de-identified, password protected excel worksheets. The data set received from SPS comprised information for purchases made between 18th July 2018, one year after the SPS announced its intention to make all prisons smoke-free in November 2018, and 30th September 2019.

We coded every purchase into one of six product types (coding by CB and initial coding reviewed by AB and KH): *tobacco-related* (including tobacco, cigarette papers, filters, lighters and cigarette rolling devices); *e-cigarette-related* (devices, e-liquids and chargers); *personal hygiene*; *food* (including sweets, snacks and beverages); *communications* (stamps, writing materials and phone cards); or *other*. There was very little ambiguity about the classification of purchases. Although the purchases had brief names in the data, these included the brand name and as there were distinct e-cigarette, tobacco, food and hygiene brands it was straightforward to classify.

We then calculated the total spend for each individual per week on all products combined and for each of these six product types. Finally, we created a combined measure of the total spend on any tobacco-related or e-cigarette-related products by each person, per week, referred to collectively hereafter as 'nicotine-related products'. These include manufactured cigarettes, rolling tobacco, papers, filters and other smoking paraphernalia and e-cigarette products such as devices, e-liquid refills, and chargers. Nicotine Replacement Therapy was not included as although it was available through the canteen purchase levels were so low as to be negligible.

For peer review only - http://bmjopen.bmj.com/site/about/guidelines.xhtml

Ethical Approval

This study was approved by the General University Research Ethics Panel of the University of Stirling ref GUEP497(A).

Patient and Public Involvement

It was not possible to involve study participants in the design, or conduct, or reporting, or dissemination plans of our research. However, the qualitative research with PiC and staff conducted as part of this wider study, explored their views and experiences of vaping in prisons before and after the implementation of smoke-free prisons and is published elsewhere [9,11].

Analysis

We conducted a series of generalised linear mixed effects models to examine changes in the total spend in the canteen per week and on the amount spent on nicotine-related products over time. These models account for the dependencies among repeated measures for each individual over time by including an individual level random intercept. The effect of the implementation of smoke-free prison policy on 'canteen' purchasing behaviour, expressed as incident rate ratios (IRRs), was estimated using an interrupted time series approach. The time series was divided into three periods:

1. the period before the removal of tobacco products from the canteen list (i.e. Period 1 29/7/2018-30/9/2018)
2. the period including the introduction of rechargeable e-cigarette products and removal of tobacco-related products (i.e. Period 2 1/10/2018-31/1/2019).
3. the period after these changes were made (i.e. Period 3 1/2/2019-31/3/2019).

The comparison of interest was between Periods 1 and 3. The estimate of effects of Period 2 versus Period 1 was of less interest as there were several changes during this time related to the imminent implementation of smoke-free policy, including the addition of two brands of rechargeable e-

For peer review only - http://bmjopen.bmj.com/site/about/guidelines.xhtml

cigarettes to the canteen list, the provision of free introductory e-cigarette packs to eligible, recorded smokers and a graded pricing structure for e-cigarette products during this period (see Brown et al [10] for more detail). Period 2 also includes the Christmas period, when canteen spending patterns are temporarily disrupted. For example, there may be two opportunities for purchase in one calendar week before Christmas and none the week after because Christmas falls on the day the canteen would usually be available. This distorts the median weekly spend for those weeks. Hence, the comparison of Period 1 versus Period 3 best captures the effect of the smoke-free policy and the introduction of rechargeable e-cigarette products in Scottish prisons.

The generalised mixed models included a factor variable for prison, a variable for week number (1 to 35), a factor variable for time period, and an interaction between the two. The distribution of the purchase data was highly skewed with lots of small purchases and relatively few high value purchases. The appropriate link function for the generalised linear model was determined by comparing the Akaike information criterion (AIC) for mixed effects Poisson, negative binomial and linear models. For all analyses the negative binomial model (log link) was associated with the smallest information loss. Marginal effects for the implementation of smoke-free prison policy were computed for all models. Marginal effects are a way of demonstrating the impact of change in one variable (pre-implementation versus post-implementation) based on a model containing features such as interactions and random effects.

We also sought to take account of smoking status and the considerable turnover in the prisoner population over time. To account for effects of turnover in prison population we conducted an analysis including only people who had made purchases in periods 1-3. In addition, in order to distinguish the impact on likely smokers and likely non-smokers amongst PiC prior to implementation, we identified those who had made any purchase of tobacco in Period 1 as 'smokers'.

For peer review only - http://bmjopen.bmj.com/site/about/guidelines.xhtml

To understand overall canteen spend, and changes in purchasing over time, we therefore conducted analyses in three different samples:

1. Group 1 - the full dataset of all purchases;
2. Group 2 - people who had purchased tobacco before implementation ('smokers' in Period 1) and made purchases through Periods 2 and 3 (i.e. those who were still imprisoned);
3. Group 3 - people who had not purchased tobacco before implementation ('non-smokers in' Period 1) who made purchases through Period 2 and 3.

As there were a very large number of observations included in the analyses, the statistical significance threshold was set at 0.01. Data management and cleaning were conducted in Python and data analysis in Stata version 15; graphs were generated in R (v 4.0.3).

<u>Results</u>

Products

Tobacco-related products pre-implementation

The total range of tobacco-related products sold in the pre-implementation period is shown in Table 1. For each product, the unit price, the total spent and the total number of units purchased within Period 1 are also presented. No information is presented for the post-implementation period, as no tobacco-related products were sold via the canteen during this period.

Of the 21 unique tobacco-related products available for purchase in the pre-implementation period, spending was heavily concentrated on a small number of products and one brand ('Brand 2') of

For peer review only - http://bmjopen.bmj.com/site/about/guidelines.xhtml

rolling tobacco accounted for 48% (185175/387608.61) of all spending on tobacco-related products in Period 1.

E-cigarette-related products

Table 2 shows the price per item, total amount spent and the quantity of items purchased for all e-cigarette-related products for sale via the prison canteen in the time periods of interest (Period 1 and Period 3). Sixteen e-cigarette-related products were available in the post-implementation period (those not yet available in Period 1 are shown as £0 spend in Table 2). Again, most purchasing was concentrated on a small number of products. The most popular product was a tobacco-flavoured e-liquids at 18mg strength ('Brand A'), which alone accounted for 53% (149795.20/280257.92) of all spending on e-cigarette-related products.

Median spend per person per week by product type

Figure 1 shows the median spend per person per week for the six purchasing categories (tobacco-related, e-cigarette-related, personal hygiene, food, communications and other). The first graph shows the composition of canteen spend for all people in the dataset (n=11944), the second graph is for 'smokers' who were present in the dataset in all three periods (n=2541) and the third is for 'non-smokers' present across all the three time periods (n=342).

The dotted lines in Figure 1 indicate the date of the introduction of smoke-free policy in Scottish prisons. The yellow shading indicates Period 2 which included the one-off provision of free e-

For peer review only - http://bmjopen.bmj.com/site/about/guidelines.xhtml

cigarette starter packs for eligible smokers; changes in purchasing patterns during the Christmas period are clearly seen in the spike in purchases in late December.

Analysis of changes in canteen purchasing behaviour

We examined the relationship between the implementation of smoke-free prison policy and weekly canteen spend by individuals for three dependent variables, i.e. individual canteen spend per week on: all products; nicotine-related products; and food and beverage products.

We examined these outcomes for: i) the full sample, i.e. all people who purchased anything from the canteen during the study period (n=11994), ii) a) 'smokers' and b) 'non-smokers' making purchases throughout the implementation period (Periods 1, 2 and 3) defined as purchasing in at least 31 of 35 weeks (n=2541). Table 3 shows results for three groups.

The results in Table 3 indicate that there was no notable change in the total spend per person per week on canteen products (IRR 0.92 99% CI 0.84 to 1.00) following implementation of smoke-free prison policy: the marginal predicted total canteen spend per person per week in the full sample in the pre-implementation period was £20.46 (99% CI 19.98 to 20.94) and the marginal predicted spend in the post-implementation period was £19.43 (99% CI 18.88 to 19.98).

The implementation of smoke-free policy was associated with a decrease in the amount spent per week on nicotine-related products or NVPs ($p<0.001$). This was evident in both the full sample (Group 1) and those who were identified as 'smokers' in the pre-implementation period and were resident in prison for the full period under study (Periods 1-3) (Group 2). The marginal predicted spend on nicotine-related products in the pre-implementation period in these 'smokers' was £9.23 (99% CI 8.35 to 10.12) and in the post-implementation period was £5.21 (99% CI 4.66 to 5.77). In the post-implementation period (Period 3) the amount individuals spent on nicotine-related products showed a slight statistically significant week by week increase.

For peer review only - http://bmjopen.bmj.com/site/about/guidelines.xhtml

There was no change in the amount spent on nicotine-related products by people designated as 'non-smokers'. That is, there is no indication people who had *not* purchased tobacco during Period 1 had started purchasing e-cigarette-related products in the post-implementation period.

In contrast, the amount spent on food and beverage products increased in the post-implementation period. This effect was only significant in Group 2 ('smokers' who were resident in the prison over for the full period under study (Periods 1-3)). The marginal predicted spend on food in the pre-implementation period by these 'smokers' increased from £8.67 (99% CI 8.23 to 9.13) to £10.24 in the post-implementation period (99% CI 9.58 to 10.90).

For peer review only

Discussion

This study has shown that on average, following the introduction of smoke-free prison policy, people in custody in Scotland spend less per week on canteen purchases for e-cigarette-related products than they previously did on tobacco-related products. The IRR for the change is 0.35 (99% CI 0.25 to 0 .50). This decline is also evident in total spend on tobacco-related and e-cigarette-related products at £393,843.45 and £280,257.92 in the pre- and post-implementation periods respectively.

Nevertheless, the amount spent on nicotine-related products still represents a large proportion of the total amount spent in the canteen by PiC, with most previous 'smokers' spending around 25% of their total spend per week in the canteen on e-cigarette-related products following implementation of smoke-free policy. This concurs with findings from qualitative interviews with PiC in Scotland. On the positive side, interviewees who were spending less on e-cigarettes post-implementation of smoke-free policy than they had previously spent on tobacco described benefits such as being able to buy healthier food from the canteen or other valued items, or being able to save money. However, there were beliefs that e-cigarettes were not affordable relative to the income for PiC with the fewest resources. Additionally, some complaints were made about the high upfront costs of

For peer review only - http://bmjopen.bmj.com/site/about/guidelines.xhtml

vaping in prison and that the e-cigarette-related products available on the canteen list at that time represented poor value for money e.g. because they were perceived as less satisfying than conventional cigarettes or as compared to costs of e-cigarette products in the community[9].

The amount spent on food and beverages by previous 'smokers' increased in the post-implementation period relative to pre-implementation The IRR for the change was 1.24 (99% CI 1.08 to 1.43). This possibly indicates snacking was used as a displacement activity following the removal of tobacco[16,17] and/or that people were able to diversify their discretionary food purchases due to saving money on nicotine-related products. Further research is required to examine differences in the breakdown of the food purchasing activity within food groups pre- and post-implementation, and whether this impacts on body mass index and other health-related indicators.

We found that although a range of tobacco products were available pre-implementation, purchasing was concentrated in a very small number of products, dominated by rolling tobacco. Post-implementation, nicotine-related purchasing was also concentrated on a narrow range of products, e.g., the most popular being the cheapest and highest strength e-liquid (18mg/ml). This possibly indicates that previous smokers were trying to maximise nicotine intake while minimising spending.

Limitations

It was not possible to link the canteen data to other sources of information about individuals (e.g. age, gender, custody status, smoking status) and we were therefore restricted to making assumption about whether individuals were smokers or not based on their purchasing patterns pre-implementation. We were also not able to link to information on smoking cessation support or the provision of prescribed NRT within the prisons' smoking cessation services.

There are local restrictions on the total amount of money PiC were permitted to spend on e-cigarettes post-implementation, set at the discretion of prison Governors. Information on the restrictions at individual prisons is not in the public domain. However, we understand that the

For peer review only - http://bmjopen.bmj.com/site/about/guidelines.xhtml

guidance to Governors on limiting e-cigarette purchasing suggested maximums far in excess of the median spend on e-cigarette products observed here.

The strengths of this study are that we have complete data from the canteen spend in closed publicly operated prisons in Scotland for a time period spanning the introduction of smoke-free prison policy. This is unique data, and we are not aware of any other studies within the UK or internationally that have been able to study the spending choices of people in custody or to evaluate the effects of smoke-free prison policy on canteen spending. This research will be important for jurisdictions considering implementing smoke-free prisons in the future.

Our conclusions are limited to the amount of money that PiC spent on tobacco and e-cigarettes pre and post implementation. This study did not aim to estimate how much nicotine was consumed and whether this differed from pre to post implementation. This would be challenging to estimate from canteen purchases given that the amount of nicotine that becomes bio-available varies enormously across products and methods of use. Effect on nicotine consumption would be an important topic for future research as it would have implications for smoking and vaping behaviour post release.

For peer review only

Conclusion

Although smoke-free policy has decreased the amount of money previous ‘smokers’ in prison spend on nicotine-related products after implementation of smoke-free policy, nicotine-related products still account for a large proportion of discretionary purchases by people in custody. Ongoing monitoring is required to understand the longer-term benefits and risks of selling e-cigarettes in prisons, including how vaping affects the personal finances and health of people in custody and influences smoking behaviour post release.

For peer review only - http://bmjopen.bmj.com/site/about/guidelines.xhtml

References

1 Graham L, Fischbacher CM, Stockton D, *et al.* Understanding extreme mortality among prisoners: a national cohort study in Scotland using data linkage. *Eur J Public Health* 2015;**25**:879–85. doi:10.1093/eurpub/cku252

2 Dumont DM, Brockmann B, Dickman S, *et al.* Public health and the epidemic of incarceration. *Annu Rev Public Health* 2012;**33**:325–39. doi:10.1146/annurev-publhealth-031811-124614

3 Hiscock R, Bauld L, Amos A, *et al.* Socioeconomic status and smoking: a review. *Ann N Y Acad Sci* 2012;**1248**:107–23. doi:10.1111/j.1749-6632.2011.06202.x

4 Carnie J, Cameron J, Downie D, *et al.* Prisoner survey 2017 16. Edinburgh: 2017.

5 Sweeting H, Semple S, Demou E, *et al.* Predictors of opinions on prison smoking bans: Analyses of survey data from Scottish staff and prisoners. *Tob Induc Dis* 2019;**17**:47. doi:10.18332/tid/109559

6 Semple S, Dobson R, Sweeting H, *et al.* The impact of implementation of a national smoke-free prisons policy on indoor air quality: results from the Tobacco in Prisons study. *Tob Control* 2020;**29**:234–6. doi:10.1136/tobaccocontrol-2018-054895

7 Piacentini L, Weaver B, Jardine C. Employment and Employability in Scottish Prisons: A Research Briefing Paper, Feb 2018. SCCJR. http://www.sccjr.ac.uk/wp-content/uploads/2018/02/Research_Briefing_Prisons_Employability.pdf. Published 2018.

For peer review only - http://bmjopen.bmj.com/site/about/guidelines.xhtml

8 NHS Health Scotland. Smoke-Free Prisons Pathway – A Service Specification Supporting People in Our Care. http://www.healthscotland.scot/publications/smoke-free-prisons-pathway-a-service-specification-supporting-people-in-our-care. Published 20189

9 Hunt K, Semple S, Demou E, *et al.* Evaluating graduated progress towards and impacts of the implementation of indoor smoke free prison facilities in Scotland. *Public Heal Res* 2021;**in press**.https://www.journalslibrary.nihr.ac.uk/programmes/phr/155544/#/

10 Demou E, Dobson R, Sweeting H, *et al.* From Smoking-Permitted to Smokefree Prisons: A 3-Year Evaluation of the Changes in Occupational Exposure to Second-Hand Smoke Across a National Prison System. *Ann Work Expo Heal* 2020;**64**:959–69. doi:10.1093/annweh/wxaa073

11 Brown A, Sweeting H, Semple S, *et al.* Views of prison staff in Scotland on the potential benefits and risks of e-cigarettes in smoke-free prisons: a qualitative focus group study. *BMJ Open* 2019;**9**:e027799. doi:10.1136/bmjopen-2018-027799

12 Sweeting H, Demou E, Brown A, *et al.* Prisoners and prison staff express increased support for prison smoking bans following implementation across Scotland: results from the Tobacco In Prisons study. Tob. Control. 2020. doi:10.1136/tobaccocontrol-2020-055683

13 Johnson C, Chaput J-P, Diasparra M, *et al.* How did the tobacco ban increase inmates' body weight during incarceration in Canadian federal penitentiaries? A cohort study. *BMJ Open* 2019;**9**:e024552. doi:10.1136/bmjopen-2018-024552

14 Johnson C, Chaput J-P, Rioux F, *et al.* An exploration of reported food intake among inmates who gained body weight during incarceration in Canadian federal penitentiaries. *PLoS One* 2018;**13**:e0208768. doi:10.1371/journal.pone.0208768

15 Scottish Government. Scottish prison population: statistics 2019 to 2020. Edinburgh: 2020. https://www.gov.scot/publications/scottish-prison-population-statistics-2019-20/pages/1/

16 Spring B, Pagoto S, McChargue D, *et al.* Altered reward value of carbohydrate snacks for

For peer review only - http://bmjopen.bmj.com/site/about/guidelines.xhtml

female smokers withdrawn from nicotine. *Pharmacol Biochem Behav* 2003;**76**:351–60. doi:10.1016/j.pbb.2003.08.008

17 Donny EC, Caggiula AR, Weaver MT, *et al.* The reinforcement-enhancing effects of nicotine: implications for the relationship between smoking, eating and weight. *Physiol Behav* 2011;**104**:143–8. doi:10.1016/j.physbeh.2011.04.043

For peer review only

Acknowledgments

We are grateful to staff at the Scottish prison service who assisted with the study and facilitated access to data. We are also grateful to the wider Tobacco In Prisons Study research team.

Funding

The study was supported by Cancer Research UK (C45874/A27016).

Competing interests

The authors have no competing interests to declare.

Contributors

AB and KH: conception of the work, acquisition of data, communication and collaboration with Scottish Prison Service, interpretation of data, and revision of manuscript for important intellectual content. CB: management and analysis of the data, interpretation of data, creating first draft of manuscript and revision of the manuscript.

Data availability statement

No additional data available.

For peer review only - http://bmjopen.bmj.com/site/about/guidelines.xhtml

Table 1 Tobacco products available for purchase from the canteen list prior to implementation of smoke-free policy

		Pre-implementation period (Period 1)	
**Product**	Price (£)	Total spend 29/7/2018-30/9/2018	Total quantity 29/7/2018-30/9/2018
Pipe tobaccos			
**Brand 1 pipe tobacco 12.5g**	2.59	83400.59	32201
**Brand 9 pipe tobacco 50g**	10.20	408.00	40
**Brand 7 pipe tobacco 25g**	6.60	3042.60	461
Rolling tobaccos			

For peer review only - http://bmjopen.bmj.com/site/about/guidelines.xhtml

**Brand 2 rolling tobacco 30g**	12.50	185175.00	14814
**Brand 5 rolling tobacco 30g**	12.90	838.50	65
**Brand 6 rolling tobacco 30g**	13.35	1174.80	88
**Brand 10 rolling tobacco 30g**	10.00	32450.00	3245
**Brand 8 rolling tobacco 30g**	11.35	66318.05	5843
Factory made cigarettes/cigars			
**Brand 3 king size 20 cigarettes**	9.60	2611.2	272
**Brand 8 20 cigarettes**	9.65	434.25	45
**Brand 4 cigar drum**	1.01	131.30	130
Paraphernalia			
**Disposable lighter**	0.10	2554.10	25543
**RYO papers**	0.10-0.26	6725.94	54937
**Filters**	0.40-0.58	2329.70	4069
**Rolling machine**	1.62	14.58	9
**Total**		387608.61	

For peer review only - http://bmjopen.bmj.com/site/about/guidelines.xhtml

For peer review only

Table 2 Spend on e-cigarette-related Products pre- and post-implementation of smoke-free policy

	Pre-implementation (Period 1)			Post-implementation (Period 3)		
**Product**	Price	Total spend 29/7/2018 - 30/9/2018	Total quantity 29/7/2018 - 30/9/2018	Price	Total spend 1/2/2019-31/3/2019	Total quantity 1/2/2019-31/3/2019
Devices						
**Brand A rechargeable device**	7.00	574.00	82	7.00	5565.00	795
**Brand B rechargeable device**	4.33	329.08	76	6.49	1090.32	168
**USB mains plug for rechargeable device**	4.00	216.00	54	4.00	1048	262
**Introductory e-cigarette pack**	0	0	0	7.10	1874.40	264

For peer review only - http://bmjopen.bmj.com/site/about/guidelines.xhtml

**Disposable e-cigarettes**	1.20	3554.20	1594	1.20	4465.80	3673
Capsules						
**Brand A capsule – tobacco flavour 18mg**	3.20	246.40	77	3.20	149795.20	46811
**Brand A capsule berrymint - 12mg**	3.20	320.00	100	3.20	44422.40	13882
**Brand A capsule menthol - 12mg**	3.20	99.20	31	3.20	24902.40	7782
**Brand A capsule strawberry- 12mg**	3.20	358.40	112	3.20	13129.60	4103
**Brand A capsule red cherry - 12mg**	0	0	0	3.20	10665.60	3333
**Brand A capsule berry mint - 6mg**	0	0	0	3.20	451.20	141
**Brand B capsule strawberry 18mg**	6.80	292.40	43	6.80	7718	1135
**Brand B capsule blackcurrant - 18MG**	6.80	244.80	36	6.80	15130	2225
**Total**	6234.84			280257.92		

For peer review only - http://bmjopen.bmj.com/site/about/guidelines.xhtml

Table 3. Results from mixed effects negative binomial regression examining changes in canteen spend pre- versus post-implementation of smoke-free prison policy

		**Total spend (£)**	**Nicotine-related products (£)**	**Food items (£)**
	Variable	IRR (99% CI)	IRR (99% CI)	IRR (99% CI)
**Full sample n=11944 (Group 1)**	Pre vs post change	0.92 (0.84 to 1.00)	0.40 (0.33 to 0.51)**	1.12 (0.99 to 1.27)
	Change in time trend	1.00 (1.00 to 1.00)	1.02 (1.01 to 1.03)**	1.00 (1.00 to 1.01)
	Pre time trend	1.00 (1.00 to 1.00)	0.99 (0.99 to 1.00)	1.00 (0.99 to 1.00)
**'Smokers' resident within a prison for more than 31 weeks (Periods 1-3) n=2541 (Group 2)**	Pre vs post change	0.91 (0.81 to 1.03)	0.35 (0.25 to 0 .50)**	1.24 (1.08 to 1.43)*
	Change in time trend	1.00 (1.00 to 1.01)	1.02 (1.01 to 1.04)**	1.00 (0.99 to 1.00)

For peer review only - http://bmjopen.bmj.com/site/about/guidelines.xhtml

For peer review only

	Pre time trend	1.00 (1.00 to 1.00)	0.99 (0.99 to 1.00)	1.00 (1.00 to 1.00)
**'Non-smokers' resident within a prison for more than 31 weeks n=342 (Group 3)**	Pre vs post change	0.87 (0.66 to 1.16)	0.08 (0.00 to 1.62)	0.80 (0.540 to 1.19)
	Change in time trend	1.01 (0.99 to 1.02)	1.18 (1.03 to 1.36)	1.01 (0.99 to 1.02)
	Pre time trend	1.00 (0.99 to 1.01)	1.03 (0.93 to 1.14)	1.00 (0.99 to 1.01)

IRR= incident rate ratio * p<0.01 ** p<0.001

Figure 1 Median weekly spend per person by product category.

For peer review only - http://bmjopen.bmj.com/site/about/guidelines.xhtml

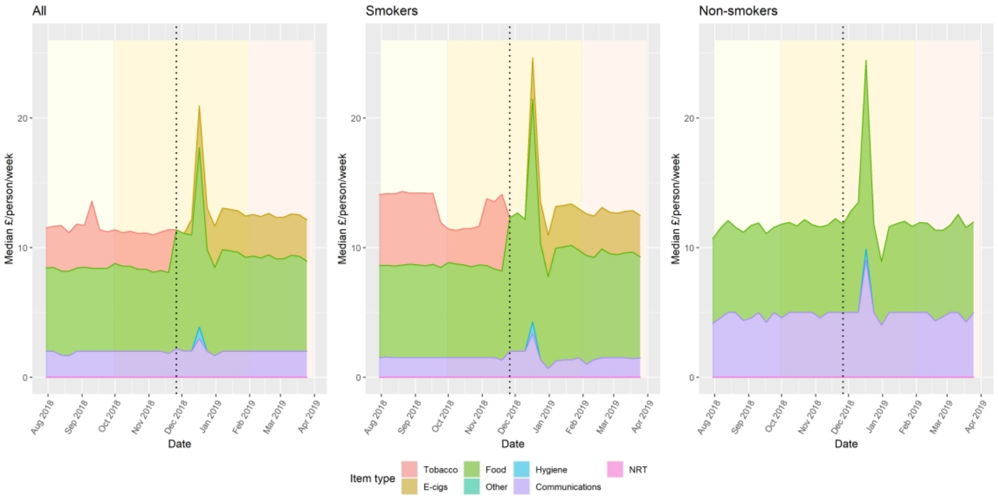

Figure 1 Median weekly spend per person by product category.

355x177mm (300 x 300 DPI)

STROBE Statement—Checklist of items that should be included in reports of ***cohort studies***

	Item No	Recommendation	Page No
**Title and abstract**	1	(*a*) Indicate the study's design with a commonly used term in the title or the abstract	1
		(*b*) Provide in the abstract an informative and balanced summary of what was done and what was found	1
**Introduction**			
Background/rationale	2	Explain the scientific background and rationale for the investigation being reported	2
Objectives	3	State specific objectives, including any prespecified hypotheses	2
**Methods**			
Study design	4	Present key elements of study design early in the paper	3
Setting	5	Describe the setting, locations, and relevant dates, including periods of recruitment, exposure, follow-up, and data collection	3
Participants	6	(*a*) Give the eligibility criteria, and the sources and methods of selection of participants. Describe methods of follow-up	3
		(*b*) For matched studies, give matching criteria and number of exposed and unexposed	
Variables	7	Clearly define all outcomes, exposures, predictors, potential confounders, and effect modifiers. Give diagnostic criteria, if applicable	3
Data sources/ measurement	8*	For each variable of interest, give sources of data and details of methods of assessment (measurement). Describe comparability of assessment methods if there is more than one group	4
Bias	9	Describe any efforts to address potential sources of bias	3
Study size	10	Explain how the study size was arrived at	4
Quantitative variables	11	Explain how quantitative variables were handled in the analyses. If applicable, describe which groupings were chosen and why	4
Statistical methods	12	(*a*) Describe all statistical methods, including those used to control for confounding	4
		(*b*) Describe any methods used to examine subgroups and interactions	
		(*c*) Explain how missing data were addressed	
		(*d*) If applicable, explain how loss to follow-up was addressed	
		(*e*) Describe any sensitivity analyses	
**Results**			
Participants	13*	(a) Report numbers of individuals at each stage of study—eg numbers potentially eligible, examined for eligibility, confirmed eligible, included in the study, completing follow-up, and analysed	4
		(b) Give reasons for non-participation at each stage	
		(c) Consider use of a flow diagram	
Descriptive data	14*	(a) Give characteristics of study participants (eg demographic, clinical, social) and information on exposures and potential confounders	4
		(b) Indicate number of participants with missing data for each variable of interest	
		(c) Summarise follow-up time (eg, average and total amount)	
Outcome data	15*	Report numbers of outcome events or summary measures over time	4, 5, 6

For peer review only

For peer review only - http://bmjopen.bmj.com/site/about/guidelines.xhtml

Main results	16	(*a*) Give unadjusted estimates and, if applicable, confounder-adjusted estimates and their precision (eg, 95% confidence interval). Make clear which confounders were adjusted for and why they were included<br>(*b*) Report category boundaries when continuous variables were categorized<br>(*c*) If relevant, consider translating estimates of relative risk into absolute risk for a meaningful time period	4 & Table 2
Other analyses	17	Report other analyses done—eg analyses of subgroups and interactions, and sensitivity analyses	6 &7
**Discussion**			
Key results	18	Summarise key results with reference to study objectives	7
Limitations	19	Discuss limitations of the study, taking into account sources of potential bias or imprecision. Discuss both direction and magnitude of any potential bias	7
Interpretation	20	Give a cautious overall interpretation of results considering objectives, limitations, multiplicity of analyses, results from similar studies, and other relevant evidence	7
Generalisability	21	Discuss the generalisability (external validity) of the study results	7
**Other information**			
Funding	22	Give the source of funding and the role of the funders for the present study and, if applicable, for the original study on which the present article is based	In electronic submission

*Give information separately for exposed and unexposed groups.

Note: An Explanation and Elaboration article discusses each checklist item and gives methodological background and published examples of transparent reporting. The STROBE checklist is best used in conjunction with this article (freely available on the Web sites of PLoS Medicine at http://www.plosmedicine.org/, Annals of Internal Medicine at http://www.annals.org/, and Epidemiology at http://www.epidem.com/). Information on the STROBE Initiative is available at http://www.strobe-statement.org.

For peer review only

For peer review only - http://bmjopen.bmj.com/site/about/guidelines.xhtml
